# Feasibility of the *Hemomindful* Program: a mindfulness-based program performed during hemodialysis

**DOI:** 10.1590/2175-8239-JBN-2024-0068en

**Published:** 2024-12-16

**Authors:** Angélica Nickel Adamoli, Bruno Nunes Razzera, Raphaele Nonnenmacher Colferai, Maitê Freitas Ranheiri, Wagner de Lara Machado, Ana Maria Pandolfo Feoli, Ana Regina Noto, Margareth da Silva Oliveira

**Affiliations:** 1 Pontifícia Universidade Católica do Rio Grande do Sul, Escola de Ciências da Saúde e da Vida, Departamento de Psicologia Clínica, Porto Alegre, RS, Brazil.; 2 Hospital de Clínicas de Porto Alegre, Serviço de Educação Física e Terapia Ocupacional, Unidade de Hemodiálise, Porto Alegre, RS, Brazil.; 3 Universidade Federal do Rio Grande do Sul, Instituto de Psicologia, Porto Alegre, RS, Brazil.; 4 Universidade Federal de São Paulo (UNIFESP), Departamento de Psicobiologia, São Paulo, SP, Brazil.

**Keywords:** Mindfulness, Hemodialysis, Kidney Failure, Chronic, Pain, Feasibility Studies

## Abstract

**Introduction:**

Recent evidence indicates that mindfulness-based programs (MBPs) improve overall well-being and the ability to cope with kidney failure and hemodialysis stressors. However, intradialytic MBPs are poorly investigated.

**Objective:**

The aim of this study was to describe the study protocol, evaluate the feasibility and perceived effects of the *Hemomindful* Program.

**Methods:**

The results presented are from a mixed-methods randomized controlled trial. Thirty-two adults with kidney failure were randomized into the *Hemomindful* Program, which consisting of 8 weekly individual sessions of 1 hour delivered at chairside during hemodialysis combined with the treatment as usual (TAU), or TAU alone. Feasibility was assessed based on retention of the study protocol, adherence to the *Hemomindful* Program, its safety, and participant satisfaction. Semi-structured interviews were conducted with participants in the intervention arm immediately following treatment. Data were analyzed using descriptive statistics and discursive textual analysis.

**Results:**

The overall rate of adherence to the study protocol was 84.38%. Among the participants in the *Hemomindful* Program (n = 16), 15 had four or more sessions (93.7%) and 12 completed the protocol (75%). Degree of importance attributed to the intervention was 8.58 (SD = 2.06) and intention to maintain the formal and informal mindfulness practices after the intervention was 6.67 (SD = 2.93) and 8.5 (SD = 2.31). The qualitative analysis indicated satisfaction with the perceived changes (greater awareness in daily activities, less reactivity, management of pain and discomfort) and the structure of the program.

**Conclusion:**

The *Hemomindful* Program showed positive indicators of feasibility, with good retention, acceptability and safety.

## Introduction

People with kidney failure undergoing regular dialysis often have a significant number of symptoms that can disrupt daily activities and reduce overall life satisfaction^1^. Numerous symptoms are documented, including fatigue, pain, low mood, dry skin, disrupted sleep, and muscle cramps, with fatigue being the most prevalent and pain the most severe^2,3^. People in hemodialysis (HD) have a lower quality of life and are more likely to develop mental disorders such as depression and anxiety^1,4^, higher levels of stress^5^, and other comorbidities, which makes adherence to treatment and survival more difficult in this population.

Due to the complexity of kidney failure, complementary interventions to hemodialysis that help reduce stressors in this population are necessary. Recent evidence indicates that mindfulness-based programs (MBPs) to improve general well-being and increase the ability to deal with kidney failure and hemodialysis stressors^6,7^ may be a promising and safe complementary therapy during hemodialysis, acting on quality of life and physical aspects of the kidney failure disease^8^.

There are many studies that indicate the positive effects of MBPs in improving the well-being of people with chronic diseases^9,10,11,12^. Mindfulness is defined as the awareness of being in the present moment, paying attention to what is happening in our body, mind, and emotions, and to all internal or external events, without judgment or resistance, with an open and gentle atitude^13^. MBPs are based on the knowledge of contemplative traditions, medicine, psychology, and education. They approach the experience with a focus on the present moment, using skills and qualities of mindfulness meditation practice to involve participants in intensive training based on experiential investigation^14^.

Some studies have assessed the feasibility and effects of traditional MBPs, such as Mindfulness-Based Cognitive Therapy (MBCT) and Mindfulness-Based Stress Reduction (MBSR), with positive results in participant quality of life and self-efficacy^15^. They are considered feasible strategies as a complementary treatment for Renal Replacement Therapy (RRT)^8,16^. However, other studies report that conventional MBPs such as MBSR and MBCT, with weekly sessions of two hours or more and long mindfulness practices (45 minutes), can decrease the adherence of people with kidney failure, especially those in HD, due to the stressful treatment at the physical, emotional, and psychological level^17^, the time demands, and travel costs^8,18^.

The feasibility and effects of MBPs adapted to the specific needs of people with a high demand of health services, such as older adults^19^ and people on RRT^6,8,17,20,21,22,23,24^, have improved. Reilly-Spong et al.^17^ adapted the MBSR model to a hybrid format of teleconference and face-to-face meetings for people in RRT and demonstrated that this model allowed for a shorter travel time and reduced travel costs. Participants suggested performing the intervention during HD to help manage the pain and anxiety that usually arise during sessions. However, few studies evaluated the feasibility of mindfulness-based interventions (MBIs) performed during HD sessions in adults^8^. These studies propose interventions with brief mindfulness meditation practices (5–30 minutes) with the support of audios^20,22,23,24^ or with guided practices using virtual reality glasses (25 minutes) with auditory and visual resources^21^ during HD. However, in these models, the shared practice and interaction between instructor and participant is minimal or nonexistent. These methodological elements are recommended in the Guidelines of Good Practice and Integrity regarding MBPs, and contextual treatment approaches are based on mindfulness and acceptance^14,25^. With the aim of expanding accessibility, reducing travel time and costs, and optimizing treatment time, the *Hemomindful* Program was developed, a new mindfulness-based stress management program during HD sessions^6^.

Robust research protocols and randomized controlled trials (RCT) to assess the feasibility and effects of MBIs in people with kidney failure are incipient and scarce^7^. Although recent studies in this population indicate that MBI are viable and safe, aspects of the protocol design, adherence to practices, and participant satisfaction using mixed method analysis need to be further explored. The present study aims to describe the study protocol of a randomized clinical trial and evaluate the feasibility and preliminary effects of the *Hemomindful* Program performed during hemodialysis.

## Method

### Study Design

The presented results are from a mixed-methods randomized controlled trial to evaluate the effects the *Hemomindful* Program combined with the usual treatment (TAU) (the intervention group - IG) compared to TAU alone (the control group - CG) concerning pain and heath conditions in people with kidney failure undergoing HD, with a follow-up of up to three months.

This study is registered in the Clinical Trials (NCT 04610593) website and was approved by the Research Ethics Committee of the Clinical Hospital of Porto Alegre (HCPA) (CAAE: 40658214.6.0000.5336 GPPG / HCPA).

### Participants

A convenience sample was selected. Participants were eligible for the trial if they were aged 18 years or over, had been receiving HD treatment for at least three months, could speak and understand Portuguese, were literate, had an interest in participating in the research, and scored the minimum cut-off point (≥ 24) of the mini-mental state examination (MMSE)^26^. Participants were excluded if they had any severe mental health disorder (diagnosed by the medical team), were medically unstable, or had previous experience with meditation or any other mind-body interventions (e.g. yoga) in the past 12 months.

### Interventions

#### Usual treatment in the hemodialysis unit

The usual care for people with kidney failure who perform HD was offered to all participants by professionals from different areas (medicine, nursing, nutrition, physical education, pharmacy) who assist them according to the individual treatment plan.

#### Hemomindful program

The program is a standardized MBP for stress management and quality of live promotion for people with kidney failure undergoing HD. The intervention was explained in a handbook and consisted of eight weekly sessions of up to 60 minutes performed individually at the bedside. It was designed considering the characteristics and needs of this population, and based on the MBSR protocol^27^, mindfulness-based relapse prevention (MBRP)^12^, and Body in Mind Training (BMT)^28^. The *Hemomindful* Program includes mainly mindfulness meditation practices, in addition to cognitive and behavioral psychology strategies and health education to deal with stress, pain and challenges related to kidney failure and HD treatment.

The program is divided into three comprehensive themes and practices aiming to develop mindfulness skills gradually and progressively, encouraging greater kindness and acceptance of experiences throughout the program. Table 1 shows the details of themes, objectives, didactic content, and practices for each session.

**Table 1:** *Hemomindful* Program

Theme	Sessions	Objective	Educational content	Practices and exercises
Awareness of the present moment	1. Be present	- Present the bases of Hemomindful; - Introduce the autopilot and make counterpoint with mindfulness; - Principles of mindfulness: intention, attention and attitude (Model IAA)^36^; - Present mindfulness as a way to recognize mental patterns; - Present the pause as a way to slow down and increase body awareness.	- Presentation of the Program; - Three C’s Invitation: Courage, Curiosity, Compassion; - Mindfulness versus autopilot; - Model IAA^36^.	- Raisin; - Pause of self-care with conscious movements. Home chores - Self-care pause with conscious movements; - Mindful eating.
	2. Inhabiting the body	- Present the practices of body scanning and conscious movements; - Increase attention to the physical, cognitive and emotional reactions to the triggers; - Present the systems of emotional regulation: threat, reward and calmness; - Show how these reactions lead us to automatic behaviors and take our attention away from what is really happening; - Present conscious movements as a form of self-care in this automatic process.	- Triangle of consciousness: body, mind, emotions; - Common practice challenges; - Emotional regulation systems.	- Body scanning; - Conscious movements. Home chores - Body scanning and conscious movements; - Pauses during the day; - Daily activity with mindfulness.
	3. Mindfulness in daily life	- Present the practices of the STOP and attentive walking sounds as possibilities to integrate mindfulness in daily life; - Awareness of the breath as an anchor always present in the experience; - Increase awareness of the use of STOP to create a space to respond instead of reacting on a daily basis.	- Intention: to live in the present moment; - Acronym STOP: Stop, Take a breath, Observe, Proceed; - Self-regulation of attention.	- Mindfulness listening; - Breathing; - STOP to breathe. Home chores - Practice of mindfulness in breathing; - STOP 3 times a day; - Changing habits: Walking with mindfulness.
Acceptance and non-reactivity	4. Welcoming pain and discomfort	- Increase awareness of bodily sensations, thoughts and emotions that tend to arise in difficult and stressful situations; - Explore primary and secondary reactions from experience; - Practice, stay and open yourself up to challenging situations such as the experience of pain, discomfort without avoiding or running away from them; - Learn skills that help to be present and not automatically give in to pressure and behave in a dysfunctional way; - Explore the discovery of needs and self-care activities they perform; - Introduce the pause with a self-passive gesture as a practice of welcoming and compassionate awareness, especially in difficult situations.	- Acceptance versus fight or flight; - Primary and secondary experience; - Mindfulness and experience with pain and discomfort; - Self-compassion.	- Surfing in emotions; - STOP in difficult situations; - List activities that help to deal with pain and discomfort; - Self-compassionate pause Home chores - Surfing the difficulties and self-compassionate pause; - STOP in difficult situations; - Modify some habit.
	5. Acceptance and skillful action	- Cultivate an attitude of curiosity, kindness and non-judgment with neutral, pleasant or unpleasant experiences that arise; - Discuss the role of acceptance in the change process, especially in relation to stress and pain; - Promote the experience of qualities of presence, rooting, dignity, strength, impermanence through metaphors; - Explore skillful self-care behaviors towards life values.	- Serenity Prayer; - Poem “The guest house”; - Calm system.	- Sounds, sensations, breathing, thoughts and emotions; - List of actions to activate the calm system; - Mountain meditation. Home chores - Sounds, sensations, breathing, thoughts and emotions; - Mountain meditation; - Change of habits - Perform an activity that activates the calm system.
	6. Thoughts are not facts	- Reduce the degree of identification with our thoughts, recognizing that we do not have to comply with or control them; - Discuss the relapse cycle of reactive behaviors and understand the role of thoughts in this process; - Explore the practice of attentive movements as an anchor for presence in moments of accelerated mental flow.	- Mental habits; - Full/empty mind and attentive mind; - Dealing with “mental monkeys”.	- Thoughts; - Conscious movements; Home chores - Conscious thoughts and/or movements; - STOP 3 times a day - Observe the “mental monkeys”; - Daily activity with mindfulness.
Changing habits and social support	7. Self-care and balanced lifestyle	- Discuss the importance of a balanced lifestyle and take care of oneself to reduce the vulnerability to relapse of reactive behaviors; - Introduce the practice of loving kindness as yet another practice of conscience and compassionate attitude towards yourself and others; - Discuss the practice of regular mindfulness as a way to maintain balance.	- Balance of lifestyle and compassion for a healthy and fulfilling life; - Awareness of activities, people and situations associated with discomfort and challenging emotions, pleasure and energy and neutrality.	- Loving kindness; - List exhausting, neutral and energizing activities; - STOP to breathe. *Home chores* - Loving kindness and others of your choice; - Energizing activity; - Report, object, drawing, music, etc ... that represents what you have learned, experienced or had value in the *Hemomindful* Program.
	8. Social support and continued practice	- Highlight the importance of support networks and the importance of balance in lifestyle, taking care of yourself to reduce stress; - Discuss the use of regular mindfulness as a means of maintaining balance; - Reflect on what was learned in the course, the reasons and strategies for continuing the practice.	- Poem “Paradox”; - Review of contents, practices and intentions; - Facilitators and barriers to continued practice; - Committed values and actions.	- Body scanning; - Concluding Meditation.

Four main techniques of formal mindfulness meditation were worked on: mindful breathing, body scanning, conscious movements, and compassionate practice, as well as brief practices to be used informally in everyday life, whether to get off autopilot or deal with challenging situations, like pain. The brief practices include: Self-care pause, STOP (stop, take a breath, observe, proceed) and Self-compassionate pause.

Each session has different moments: Checking (how the participant is doing at that moment); mindfulness practice; inquiry of the experience; psychoeducation; home chores; and closure check. The mindfulness concept and skill are developed from the experience with exercises and 5 to 20-minute audio practices by the instructor, in which the participant and instructor practice together with the support of shared headphones and different guided practices. In each session, informative materials on the topic of the session, exercises and a diary were distributed to participants to describe their experiences with the practices during the week.

In addition, participants were encouraged to perform formal and informal practices at home and on other days at least once a day. To deepen their practice, they received audios of guided practices recorded by the instructor on CD or digital format. At the end of the intervention, the information materials used in each session were compiled in a manual to support continued practice.

The *Hemomindful* Program was conducted by a certified mindfulness instructor trained in the MBRP and BMT protocols, and consolidated meditative practice, and with more than ten years of experience. The instructor was part of the multi-professional team at the HD unit where the study was conducted.

Notes were made in field diaries and audio recording of all sessions were taken during the intervention to document participant receipt of treatment and any deviations from the intervention checklists.

### Procedures

**Figure 1:**
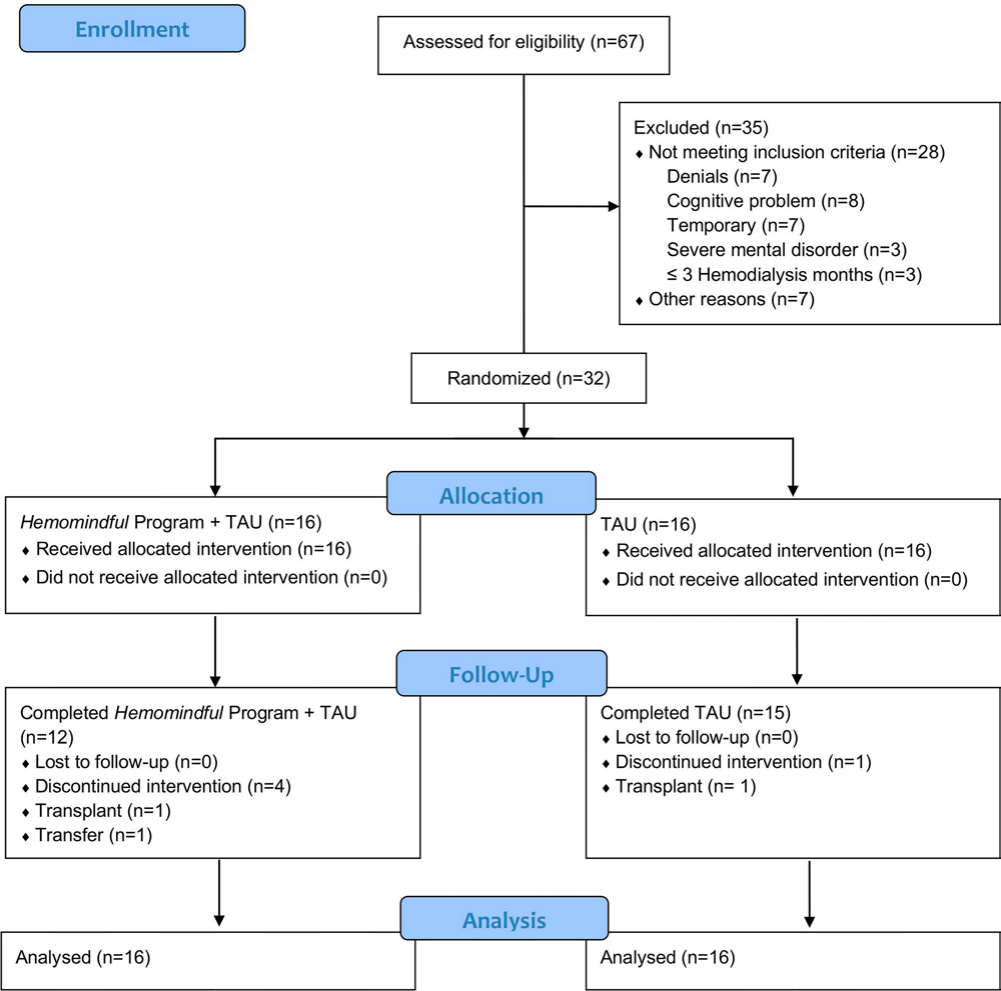
Study flowchart.

The screening involved checking the list of individuals seen at HD Unit and identifying those who met the eligibility criteria using information from electronic medical records. The project coordinator then explained the research in detail to each potential participant, informing about the voluntary nature of participation, and that they could leave the study at any time with no effect on their treatment. Those who agreed to participate in the study signed the informed consent form. A final screening and other data collection phases were conducted face to face by trained interviewers. After collecting baseline data, the intervention group (IG) participants received information about the *Hemomindful* Program, while the control group (CG) participants remained in the TAU routine, without receiving additional instruction, and were reminded that after completing the study they could undertake the program if they wished. Participants were not blinded to group status. The sample size was estimated based on previous research and on differences between groups at a significance level of 5% with a power of 85% and an effect size of Cohen d = 1.0. This required the enrollment of 30–40 participants (15–20 participants per group). Randomization was performed using the stratified 1:1 random allocation method. During the recruitment period (April –June 2019), 32 participants were enrolled, with 16 individuals randomized to the *Hemomindful* program (IG) and 16 individuals to the TAU (CG) (Figure 1). The randomization sequence was created with the R language statistical environment using the m*inDiff* package^29^ by a researcher who was neither involved in the evaluations nor in the selection of participants.

Note – TAU: Treatment as Usual.

Results related to the effects of the program included self-reported quantitative and qualitative measures and levels of inflammatory biomarkers measured at baseline, 8 weeks, and 3 months after the intervention (January – May 2020). Pain, assessed by the Profile of Chronic Pain: Screen - PCP:S scale^30^, was the primary outcome of the study. This study will focus on the study design, feasibility outcomes, and effects perceived by participants of the *Hemomindful* program, while the complete effectiveness outcomes of the program comparing CG and IG will be reported elsewhere.

### Outcome Measures

The profile of the participants was assessed through a questionnaire with sociodemographic and clinical information, which was developed by the research team to obtain information, such as age, race, education, economic level of the participants according to the Brazilian Economic Research Association (ABEP)^31^ criteria, comorbidities, treatment time, and previous meditation.

The MMSE was used to track cognitive impairments^26^. The MMSE is composed of questions that correlate in five dimensions: concentration, language/praxis, orientation, memory, and attention, with a maximum score of 30 points. The 23/24 cut-off point is the most commonly used. This instrument was applied and validated for the Brazilian context, demonstrating high sensitivity and specificity in detecting cognitive impairment^26^.

Feasibility was assessed by the number of participants who completed the protocol, adherence to the *Hemomindful* Program, implementation, safety, and satisfaction with the treatment. Retention to the study protocol was assessed using the primary data of all participants at the beginning of the study.

Adherence to the *Hemomindful* Program was measured using the weekly records of the research team in the field diaries about participation in each session and frequency of formal and informal mindfulness practice at home reported by the participants (number of days per week). Implementation and safety were assessed based on field diary records of adverse events observed by the research team.

Satisfaction with the treatment and experience of the participants were assessed immediately after completion of the intervention (8 weeks post-randomization) through a semi-structured qualitative individual interview using a questionnaire for reflections on the *Hemomindful* program, adapted from Bowen and collaborators^12^, with closed-ended questions (answers on a Likert scale from 0 to 10; “10 = very likely/very important”) and open-ended questions addressing topics such as the degree of importance of the program, perceived changes, the intervention during HD sessions, the continuity of mindfulness practices after the intervention, and suggestions for improving the intervention for future use. The interviews were digitally recorded, anonymized, and transcribed verbatim for analysis. The interview took between 15 and 30 minutes.

### Data Analysis

For quantitative data, descriptive statistical analysis was performed. Continuous variables are expressed as mean ± standard deviation. Categorical variables are expressed using frequency and proportion. Comparisons were evaluated by Pearson’s χ^2^ test or Student’s *t*-test. All numerical analyses were conducted in SPSS version 23. The qualitative data about treatment satisfaction and experience of the participants in the *Hemomindful* Program was literally transcription and the discursive textual analysis method was used, consisting of three stages: unitarization, categorization, and analysis of the categories^32^. The analysis was performed by two independent coders. Coding was based on a semantic approach in which the codes are derived from the explicit meaning of the data. They then independently coded the data on the main themes and theme categories. The categorization of the data was completed through an interactive process and a consensus approach to disagreements.

Quantitative descriptive analysis of the characteristics of the sample were carried out for the CG and IG groups, while the qualitative analysis was only carried out for the IG group.

## Results

### Baseline Characteristics

Of the 67 potential volunteers, 32 were selected (28 being ineligible and 7 declined to take part) (Figure 1). The average age was 55 years (min 24, max 84, SD = 15.6), the majority were women (n = 19; 59.4%), blacks and browns (according to self-reported skin color) represented 50% of the sample (n = 16), and most lived with family members (n = 29; 90.6%). Nineteen had primary or higher education (59.4%), 15.6% (n = 5) were in paid employment, and the majority of participants were in economic class C (n = 15; 46.8%). The duration of HD therapy, on average, was 6.28 years (min 0.3, max 20.3, SD = 5.67), and participants had an average of 3.94 comorbidities (SD = 2.32) associated with kidney failure, with pain being the most prevalent (n = 19; 59.37%). The groups were similar in terms of socio­demographic and clinical characteristics (Table 2).

**Table 2:** Baseline characteristics of participants randomized to the *Hemomindful* Program or Usual As Treatment (TAU) (n = 32)

Variables	*Hemomindful* (n = 16) Mean ± SD	TAU (n = 16) Mean ± SD	
	N (%)	N (%)	*Satistics*
**Age** (years)	52.50 ± 16.16	58.25 ± 14.9	*t* ^a^ = –1.04, *p* = .304
**Gender**			*χ* ^2b^ = 1.16, *p* = .280
Men	8 (50)	5 (31.2)	
Women	8 (50)	11 (68.8)	
**Race**			*χ* ^2b^ = 1.06, *p* = .301
White	7 (43.8)	8 (50.0)	
Black	6 (37.5)	6 (37.5)	
Brown	2 (12.5)	2 (12.5)	
Yellow	1 (6.2)	0 (0.0)	
**Live with**			*χ* ^2b^ = .36, *p* = .544
Alone	2 (12.5)	1 (6.2)	
With Family	14 (87.5)	15 (93.8)	
**Education**			*χ* ^2b^ = .58, *p* = .443
Incomplete elementary school	7 (43.8)	6 (37.5)	
Complete elementary school	2 (12.5)	3 (18.8)	
Incomplete or complete high school	5 (31.2)	4 (25.0)	
Incomplete or complete higher education	2 (12.5)	3 (18.7)	
**Employment Situation**			*χ* ^2b^ = .23, *p* = .238
Paid work	3 (18.8)	2 (12.5)	
Retirement	13 (81.2)	14 (87.5)	
**Economic level**			*χ* ^2b^ = .73, *p* = .391
A	1 (6.2)	1 (6.3)	
B1–B2	5 (31.3)	7 (43.7)	
C1–C2	8 (50.0)	7 (43.7)	
D–E	2 (12.5)	1 (6.3)	
**Treatment**			
Years of hemodialysis	6.85 ± 6.22	5.72 ± 5.2	*t* ^a^ = .55, *p* = .581
Comorbidities	4.69 ± 2.58	3.19 ± 1.83	*t* ^a^ = 1.89, *p* = .068
Use of psychotropic medication	9 (56.3)	5 (31.3)	*χ* ^2b^ = 3.27, *p* = .071

Note – SD: Standard deviation; Data presented as mean ± SD or n (%). TAU: Treatment as Usual; *Hemomindful: Hemomindful* Program.

*^a^Student’s *t* test.

^b^Pearson’s chi-squared test.

### Retention in the Protocol and Baseline Data

None of the participants (n = 32) left the protocol (100% retention). Baseline data was collected from all participants at the start of the study. In the IG group, there were four drop-outs over the 8 weeks (25%) due to “tiredness” and “lack of motivation to practice” (n = 1), transfer to another treatment unit (n = 1), transplant (n = 1), and death (n = 1). In the CG group, one volunteer (6.25%) was lost due to kidney transplantation. The general rate of retention to the study protocol was 84.38%.

### Adherence, Implementation, and safety of the *Hemomindful* Program

The participants of the IG group (n = 16), 93.75% (n = 15) performed four or more sessions of the *Hemomindful* Program and 75% (n = 12) completed the eight scheduled sessions. Some sessions had to be rescheduled because the participant was unwell at the scheduled time (e.g. nausea, vomiting, complications in the HD access), and were held on another day of HD planned for the participant.

During the study, all the participants provided data on mindfulness practice at home, 12 provided complete data for weeks 1-8 (intervention period) and four participants provided partial data that were used to estimate their average days of practice at home per week. Participants who completed the eight sessions of the *Hemomindful* Program (n = 12) reported an practicing formal mindfulness for an average of 1.88 (SD = 1.97) days and a total of 13.17 minutes (min 1, max 49; SD = 13.75) and a short or informal weekly practice of 3.79 (SD = 2.15). The program lasted 26.50 days (min 7, max 49; SD = 15.07) (days between the first and eighth sessions). The average weekly involvement with mindfulness practices at home was different according to the week evaluated, with more frequent formal practices at weeks 3 and 6 and informal practices at weeks 1 and 3 (Table 3).

**Table 3:** Mean frequency (in days per week) of formal and informal *mindfulness* practice at home and throughout the period

Week	N	Formal practices		Informal practices	
		Mean	SD	Mean	SD
Week 1	16	1.94	2.26	5.0	3.09
Week 2	16	0.88	1.92	2.5	3.20
Week 3	15	2.0	1.89	4.73	3.01
Week 4	14	1.87	2.03	3.87	3.20
Week 5	13	1.62	2.14	2.23	3.24
Week 6	12	2.38	2.39	3.46	3.25
Week 7	12	1.33	2.06	2.0	3.13
All period	12	13.17	13.75	26.50	15.07
Total weekly average	12	1.88	1.97	3.79	2.15

Note – SD: Standard deviation.

The participants received 13 audios of mindfulness practices, ranging from 3’27” to 16’50”, totaling 147 minutes of guided practice. The main formal practices worked on were self-care pauses (n = 12), breathing (n = 10), and mountain meditation (n = 10). The informal practice of pausing or STOP (Stop, Take a breath, Observe, Proceed) to breathe was the most prevalent, mentioned by all participants (n = 16), and the practice of mindfulness when eating and conscious movements were mentioned 25 and 8 times, respectively, during the program. Participants also reported carrying out informal mindfulness when playing with their children and grandchildren, bathing, brushing their teeth, and gardening. They also reported changing their habits, such as watching less television, decreasing the use of cell phones, observing nature, painting, and taking more care of their appearance, among others.

During the study, no participant reported serious or unexpected adverse events. Some participants noticed mild and/or moderate side effects during the intervention, such as increased pain perception in conscious movement practices or when invited to investigate the experience of pain (n = 3) and anxiety (n = 3) during practice.

### Satisfaction and Experience with the Treatment

Participants who completed the *Hemomindful* Program (n = 12, 75%) rated it as “very important”, with an average score of 8.58 (SD = 2.06). When asked why they attributed the chosen value, they mentioned that the program was important for different reasons, such as: “reducing stress”, “learning new things”, “spiritual benefit”, “tranquility”, “willpower”, “more mood”, “energy”, “disposition”, “learn”, “change for the better”, “know yourself better”, “enjoy the good things”. Among the participants who attributed lower values to the importance of the program (n = 2), one was unable to answer why, and another said that the program did not match his expectations.

Regarding the degree of interest in mindfulness at the end of the intervention, the participants had an average score of 8.5 (SD = 2.15) on a Likert scale from 0 to 10. On the probability of continuing to perform formal mindfulness practices (e.g., mindfulness of breathing, body scanning, conscious movements, among others) and informal ones (STOP, eating and walking with mindfulness, among others) they indicated an average score of 6.67 (SD = 2.93) and 8.5 (SD = 2.31), respectively.

From the answers to qualitative open-ended questions about their experience of the *Hemomindful* Program, two broad themes emerged: the participants’ perception of perceived changes and comments on the structure of the program, subdivided into three categories each, with representative quotes shown in Table 4.

**Table 4:** Qualitative analysis of satisfaction and experience with the *Hemomindful* Program

Categories	Representative speeches of the participants
**Theme 1. Perceived changes**	
Mindfulness in everyday life.	*“For example, taking a shower. I try to be quicker in the bath, because I pay close attention to avoid falling, I need to pay close attention to getting dressed. I try to pay attention to what I’m doing so as not to leave my mind empty and not to do things quickly so as not to be a problem”. (P2)* *“The thing of eating habits changed, before I didn’t eat fruit now I already eat fruit, so it changed a lot in my health, because I learned to eat better, to have more patience also to eat, because before I tried to do everything running, not now, I try to do everything calm”. (P12)* *“Ah… how can I tell you… it’s… posture. Yes, posture has changed, observation”. (P1)* *“It changed something during my day or during the period that I am going to say the prayers, I fit what I learned and further increases my practice.” (P2)* *“It changed a little, it changes, everything changes, the memory of everything you saw, the answers, the things you heard, everything changes”. (P5)* *“My behavior, thinking, has changed a lot. In relation to the family, daughter, my behavior has changed for the better”. (P6)*
Non-reactivity.	*“Ah, the self-control of my desires, practical desire, water, food, things like that. I think well, if I really need it, if not, I can wait a little longer to drink, this I have done right. I’m trying, holding on, trying to cope with thirst”. (P8)* *“I found it more valuable to try to master the decision, that explosive decision, that decision at the moment, that I thought was cool, it was something I’m trying to do, step back and take a deep breath”. (P8)* *“I learned how to control myself, which before I did not control myself, for me it was very good, to have done this, because before I was irritated a lot, and now I learned this, to control myself, I have to wait a while, then I also think to answer, I didn’t think of that before”. (P12)* *“(...) my way of acting has changed more, my new way is as follows, I first think and then do it, at first I no longer thought, I went there and did it and everything went wrong, it improved the way I acted a lot with the body huh”. (P4)* *“Yes, that I’m too agitated I stop, I think, I breathe, right? and come back calmly”. (P1)* *“It changes the way of thinking, the way of acting, I do my things more willingly”. (P10)*
Dealing with pain and discomfort.	*“I thought it was a learning experience for us, you know, it’s a self-help (...) if today I’m not very nice and I don’t want to be in this situation, then I can try to improve, and then it really helps”. (P10)* *“The bad things go, like it doesn’t even… go away, go away, that also stimulated me a lot… it’s like thinking it won’t work, no, it will. I had bad thoughts, I was negative, practice taught me a lot”. (P11)* *“Oh, because it was possible for us to avoid stress, you see that you are wrong ... (silence)”. (P1)* *“The coolest, because things that happened back there I couldn’t cope with and after I started doing it, I managed to make everything change for the better”. (P6)* *“I learned that we can (...) get out of that situation”. “Because sometimes when I’m really down I go back and take a look at the book, I turn to the book of practices”. (P10)* *“Physical I don’t think so, but emotional has a good meaning. I don’t get so nervous anymore, I don’t think, I don’t care much”. (P3)* *“I actually succeeded with the emotional thing, I often came here excited about the problems at home and then the study touched my body and mind. How many times did I get here bad, even thinking about giving up everything, but I was going, I was going, I was going, until the end”. (P4)* *“(...) before I didn’t know how to deal with my discomfort and today I already know. I think about the practices that I did, I think about them and I take a deep breath and the discomfort goes away”. (P6)* *“No. Because if I think about the arm, it hurts! Then it hurts!”. (P7)* *“I realized, because I relax a lot, I manage to relax”. (P2)* *“I try to understand better where the pain is, I try, how it is ... to go inside the pain to see what I can do. It is different, before I had pain, I had symptoms and took medicine, sometimes I was irritated. When we are not in control of something it is very frustrating, it is very frustrating we try and fail, that very bad feeling of helplessness”. (P8)* *“Discomfort is more emotional, with this big machine, like a nuisance. But little has changed with the practices”. (P8)* *“(...) My life has now come down to a machine, not going to Capão (beach), I might as well go there on weekends, see my children there. I can’t, you have to come here, sometimes it irritates you, so it’s a way of dealing with pain, emotional pain, physical pain ... it’s very strange”. (P2)* *“For me it remains the same”. (P9)* *“Yes, I noticed a change, because before I complained about my pains and now I no longer complain, now I can face the pain without complaint and it has decreased a lot”. (P12)*
**Theme 2. Structure of the** *Hemomindful* **program**	
Better use of HD time.	*“Great, 10, because the hours pass quickly and you don’t get agitated here”. (P1)* *“I thought it was cool, I don’t know, because it helps a lot not to be anxious about wanting to get out of here fast”. (P3)* *“I thought it was really cool, because it’s something that is helping, it helped me a lot, I thought it was really cool.” (P6)* *“Well, why at least you stay here for 4 hours, sometimes I feel good, sometimes I feel bad, but you’re there wanting everything to come out well, here are those cool words from the teacher, you like that”. (P2)* *“I have more patience now to wait all these hours, which used to irritate me a lot, and not now”. (P12)* *“Very good, because it is a time well spent”. (P8)*
Materials, practices and staff.	*“I learned everything, despite the memory, the things she says and the ringing of the bell”. (P5)* *“Well, that bell was really good, those headphones, she talking...”. (P2)* *“Everything was important, all lectures (...) there is the one about the mountain, the monkey, the stone.” (P5)* *“Because sometimes when I’m too low I take a look at the book, I turn to the book of practices”. (P10)* *“I think that the practices could be more objective and less practice time (...) and their format did not need to be so equal, they could be more diversified, because it always starts with the same thing and ends with the same thing, that the whole practice didn’t take more than an hour”. (P8)* *“They are good, they were well used, they help, they give attention, the words, talking to us, they are different people is good, very good”. (P2)* *“That it was very good, the teacher was very nice, she has all the patience with you, and she feels so good to do it, she made us feel so good, she wants us to be well too, I don’t know how to express myself ...”. (P1)* *“The way of teaching and what I learned I put in my head and in my heart and I am still going on, it is still inside me, it did not come out, it didn’t leave, just like that day you asked me a question and I understood what it was”. (P4)* *“All eight programs were good.” (P4)* *“I use the booklet, I’m a little lazy like that, you know.” (P10)* *“There was nothing to hinder me, on the contrary, I was super helped”. (P12)*
Continuity of the program.	*“It was very good and another thing, the practice of this work that started to be done and cannot stop, because it brings a lot of benefit to us, and we do it like washing our minds and not letting it overload, we have to keep it to relax”. (P2)* *“I would like everyone to stop, think and do it, because that is very good, it helps a lot”. (P12)* *“I think I should continue, to do with other people, just as it helped me, it will help many other people who are really in need, keep doing this project that will help a lot of people”. (P6)*

Note – P: Participant.

When analyzing perceived changes, three categories emerged: mindfulness in daily activities, non-reactivity, and management of pain and discomfort. Participants reported that the program contributed to increased mindfulness in daily activities, helping them to be more present and aware when carrying out daily activities such as bathing, eating, walking, and dressing. In addition, they noticed changes in interpersonal relationships and in the way they related to the demands of everyday life, adopting a posture of greater observation and tranquility. As for non-reactivity, many participants perceived a change when relating to internal and external experiences, especially in relation to unpleasant experiences. The reports expressed how they learned to “step back and take a deep breath” to make conscious choices instead of simply reacting automatically to the experience. Some participants commented on the use of mindfulness practices to deal with the challenges of kidney failure and RRT, such as HD time, water restriction, and dietary changes imposed by treatment. Regarding the management of pain and discomfort, participants observed changes in dealing with these stressors. They reported that practicing mindfulness helped them to deal with physical sensations and unpleasant thoughts and emotions, and to make choices that helped their self-care and the regulation of their emotions. Attitudes such as recognizing and exploring the pain without trying to control it, as well as thoughts and emotions of “letting go” and using resources such as STOP to breathe and perform conscious movements were mentioned. Pain reduction and positive changes on an emotional level were reported. One participant reported that her pain increased when she observed the sensations of her body, and another said that she did not notice any pain-related changes.

On the program structure theme, the following categories emerged: better use of HD time; materials, practices, and staff; and continuing the program. The better use of HD time was mentioned by 50% (n = 6) of the participants who completed the intervention. They considered that, in addition to feeling the time passing faster, they could use the time to learn to practice mindfulness and deal with the anxiety and irritation that arise during HD. In respect of materials, the participants appreciated the use of some items during the intervention, such as the bell, headphones, and the practitioner’s manual. Most of the participants were satisfied (or “very satisfied”) with the mindfulness practices, with one participant suggesting that formal practices could be more objective and diverse. The majority were also satisfied with the content and the length of the sessions of the *Hemomindful* Program, with only one participant suggesting that the sessions could take less than an hour. Most participants reported that the presence and attitude of the research team and investigation of experiences by the instructor enabled good interaction and exchange of experiences. The majority of the participants reported that they would (or were likely to) continue the program because they considered it to be useful and it provided important learning opportunities.

## Discussion

Our study shows that the *Hemomindful* Program presented positive indicators of feasibility. The retention rate in the *Hemomindful* Program was high compared to results from previous studies with people with kidney failure^17,20,21,23,24^, with 93.75% of the participants having four or more sessions and 75% completing the eight sessions of the program. Thomas et al.^24^ had a 71% retention rate in an MBI with brief mindfulness practices during HD compared to TAU only. In the feasibility study of an adapted MBSR^17^, carried out outside the context of HD treatment, with two face-to-face and six sessions by teleconference, 84% of participants had three or more sessions and only 36% completed the program. Other studies that investigated the feasibility and effects of MBI in reducing stress in people with kidney failure, carried out outside the of context using traditional group protocols, such as MBCT^15^ and MBSR^33^, did not clearly report the participants’ adherence rates. Dropouts in our study were due to similar reasons as in previous studies: tiredness, lack of interest in carrying out the program, transfer to another unit, transplantation, health deterioration, and death^15,17,24^.

Participants reported practicing formal mindfulness at home one or two days a week on average. Only one study on MBI for people with end-stage renal disease investigated their involvement with formal mindfulness practices at home, and the participants reported practicing three times a week on average^24^. Ribeiro et al.^19^ assessed adherence of older people (50-80 years) with multiple comorbidities to mindfulness practice during and after a 6-week intervention performed individually and found no association between duration of engagement and changes in psychological state and quality of live. An RCT that evaluated the effects of MBCT in preventing depression relapse showed an inverse association between the duration of formal mindfulness practice performed at home and the probability of relapse to depression^34^.

Informal mindfulness practices 4 or 5 days a week were reported by all participants. Other studies also showed greater adherence to informal mindfulness practices^33,34,35^. Research conducted with people practicing mindfulness for a year or more has shown that the frequency of informal practice is more important for increasing psychological well-being and flexibility than formal practice^34^.

Satisfaction with the *Hemomindful* Program was evidenced through quantitative and qualitative results, with the participants attributing a degree of importance (8.5) similar to that in another study performed during HD sessions (8.3)^24^ and better than that of a study with MBCT adapted for teleconferencing (8.0)^17^. Our results indicate that participating in the *Hemomindful* Program contributed to the extension of the participants’ mindfulness practices beyond the context of HD, especially when performing activities of daily living such as eating, dressing, walking, talking with friends and family, among others. Our results point to a reduced reactivity to everyday situations related both to factors associated with the kidney failure treatment, such as food and water restrictions, or use of medications, and to various personal and family situations. The habitual way of the human being is the *“way of doing”*, of solving problems with the aim of reducing the distance between where we are and where we would like to be. This mode is effective for solving external problems, but when it comes to solving internal problems, it only creates more problems, because when trying to search for causes and reasons why things are not as we would like them to be, thoughts of analysis, judgment and comparison often arise that can generate guilt, anxiety and stress^12,13^. The ability of mindfulness to consciously perceive internal and external experiences in an attentive, gentle, curious and non-judgmental way is the opposite of the automatic, reactive mode^12,36^.

A better management of pain and discomfort was one of the changes perceived by the participants. Mindfulness is an important construct in the field of chronic pain in adults, as it emphasizes a non-judgmental attitude towards the experience of the present moment, carefully observing - rather than reacting automatically – to the physical sensations, emotions and thoughts that are present^27,37^. MBPs are considered a promising complementary treatment for people suffering from pain such as headache^38^, back pain^35^ and fibromyalgia^39^. Studies suggest that practicing mindfulness regularly can lead to changes in pain acceptance and intensity perception^39,40^.

Few studies have evaluated the feasibility of MBI in the context of HD, which also proved to be feasible^21,24^. Studies are investigating the feasibility of an adapted intervention of the *Hemomindful* Program that follows the international recommendations on MBPs for clinical and educational research, with mindfulness practice as a central element, with solid conceptual bases and adequate psychoeducational support^14,25^. The qualitative analysis highlighted different parts of the program, such as the role of the instructor and research team, the way of teaching, the way instructors asked questions participants about their experiences, and gave them the opportunity to talk to other people. The importance of the experience of the investigation process for the development of mindfulness skills is described in other studies as an essential element in learning and the process of change^12,14^.

The use of different educational materials during the intervention were also valued by the participants. These resources are used to increase the focus during practice and facilitate learning^6,34^, and the decision to use them was based on difficulties reported in other studies in the context of HD^24^ and recommendations from studies with people with kidney failure outside the context of HD^17,18^.

Another important aspect is that the *Hemomindful* Program was conducted by an instructor who was part of the dialysis staff, which may have contributed to intervention adherence, given that MBPs developed by trained and certified care team professionals usually have better adherence results^40^.

### Limitations and Future Directions

Our study has some limitations, which should be noted. First, the study used a convenience sample of patients from a single HD unit, which may have caused some bias in the results. Second, the *Hemomindful* Program is a multifaceted program composed of different mindfulness practices and psychoeducational contents and techniques, making it difficult to discern the relative importance of each element of the program. Future studies should, therefore, carry out RCTs with active control groups to identify the influence of each element of the program. Finally, adherence to formal and informal mindfulness practices was measured based on participant reports, which could result in an under- or over-notification of the practice performed. We consider it is important to improve ways of measuring adherence to formal and informal mindfulness practices and to evaluate the profile of people who adhere to certain practices. More research is needed to qualify the current intervention protocol and identify the mechanisms underlying the observed positive preliminary effects.

## Conclusions

The *Hemomindful* Program was found to be a complementary intervention to TAU based on the positive indicators of feasibility and good retention, acceptability and safety. In addition, preliminary qualitative results were presented on the impact on the management of pain and discomfort associated with the treatment of HD and adherence to behaviors related to treatment, health and well-being of the *Hemomindful* Program participants. According to our results, these benefits not only help with pain management, but also contribute to improving general well-being during HD treatment. If further studies support these effects, the *Hemomindful* Program can be useful in a broad spectrum of health conditions to help reduce pain-related suffering.
