# Supplementary material for: Feasibility of the Hemomindful Program: a mindfulness-based program performed during hemodialysis
Source: J Bras Nefrol. 2024 Dec 16;47(1):e20240068. doi: 10.1590/2175-8239-JBN-2024-0068en (PMC11648740; doi:10.1590/2175-8239-JBN-2024-0068en)
Supplement: Translation

# Translation: Viabilidade do Programa *Hemomindful*: um programa baseado em *mindfulness* realizado durante a hemodiálise

**DOI:** 10.1590/2175-8239-JBN-2024-0068pt

**Authors:** Angélica Nickel Adamoli, Bruno Nunes Razzera, Raphaele Nonnenmacher Colferai, Maitê Freitas Ranheiri, Wagner de Lara Machado, Ana Maria Pandolfo Feoli, Ana Regina Noto, Margareth da Silva Oliveira

**Affiliations:** 1 Pontifícia Universidade Católica do Rio Grande do Sul, Escola de Ciências da Saúde e da Vida, Departamento de Psicologia Clínica, Porto Alegre, RS, Brasil.; 2 Hospital de Clínicas de Porto Alegre, Serviço de Educação Física e Terapia Ocupacional, Unidade de Hemodiálise, Porto Alegre, RS, Brasil.; 3 Universidade Federal do Rio Grande do Sul, Instituto de Psicologia, Porto Alegre, RS, Brasil.; 4 Universidade Federal de São Paulo (UNIFESP), Departamento de Psicobiologia, São Paulo, SP, Brasil.

**Keywords:** Mindfulness, Hemodiálise, Crônica, Insuficiência Renal, Dor, Estudos de Viabilidade

## Abstract

**Introdução:**

egundo evidências recentes, os programas baseados em mindfulness (PBM) melhoram o bem-estar geral e a capacidade de lidar com a insuficiência renal e os fatores de estresse na hemodiálise. Entretanto, PBM intradialíticos são pouco investigados.

**Objetivo:**

Descrever o protocolo do estudo, avaliar a viabilidade e efeitos percebidos do Programa *Hemomindful*.

**Métodos:**

Os resultados apresentados são de um ensaio clínico randomizado de métodos mistos. Trinta e dois adultos com insuficiência renal foram randomizados para o Programa *Hemomindful*, que consiste em 8 sessões individuais semanais de 1 hora realizadas na cadeira durante a hemodiálise, combinadas com o tratamento usual (TAU), ou TAU isolado. A viabilidade foi avaliada conforme retenção do protocolo, adesão ao Programa Hemomindful, sua segurança e satisfação do participante. Realizamos entrevistas semiestruturadas com participantes do braço de intervenção imediatamente após o tratamento. Dados foram analisados utilizando estatísticas descritivas e análise textual discursiva.

**Resultados:**

A taxa geral de adesão ao protocolo foi de 84,38%. Entre os participantes do Programa (n = 16), 15 realizaram quatro ou mais sessões (93,7%) e 12 completaram o protocolo (75%). O grau de importância atribuído à intervenção foi 8,58 (DP = 2,06) e a intenção de manter as práticas formais e informais de *mindfulness* após a intervenção foi 6,67 (DP = 2,93) e 8,5 (DP = 2,31). A análise qualitativa indicou satisfação com as mudanças percebidas (maior consciência nas atividades diárias, menor reatividade, manejo da dor e desconforto) e com a estrutura do programa.

**Conclusão:**

O Programa *Hemomindful* apresentou indicadores positivos de viabilidade, com boa retenção, aceitabilidade e segurança.

## Introdução

Pessoas com insuficiência renal submetidas a diálise regular frequentemente apresentam um número significativo de sintomas que podem prejudicar as atividades diárias e reduzir a satisfação com a vida em geral^1^. Diversos sintomas estão documentados, incluindo fadiga, dor, mau humor, pele seca, sono prejudicado e cãibras musculares, sendo a fadiga o sintoma mais prevalente, e a dor, o mais severo^2,3^. Pessoas em hemodiálise (HD) apresentam uma qualidade de vida inferior e são mais propensas a desenvolver transtornos mentais, como depressão e ansiedade^1,4^, níveis mais elevados de estresse^5^ e outras comorbidades, o que dificulta a adesão ao tratamento e a sobrevida dessa população.

Devido à complexidade da insuficiência renal, são necessárias intervenções complementares à hemodiálise que ajudem a reduzir os fatores de estresse nessa população. Evidências recentes indicam que os programas baseados em *mindfulness* (PBM) para melhorar o bem-estar geral e aumentar a capacidade de lidar com a insuficiência renal e os fatores de estresse na hemodiálise^6,7^ podem ser uma terapia complementar promissora e segura durante a hemodiálise, atuando na qualidade de vida e nos aspectos físicos da insuficiência renal^8^.

Existem muitos estudos que indicam os efeitos positivos dos PBM na melhora do bem-estar de pessoas com doenças crônicas^9,10,11,12^. O termo *mindfulness* é definido como a consciência do momento presente, prestando atenção ao que está acontecendo em nosso corpo, mente e emoções, bem como a todos os eventos internos e externos, sem julgamento ou resistência, com uma atitude aberta e gentil^13^. Os PBM são fundamentados no conhecimento de tradições contemplativas, medicina, psicologia e educação. Eles abordam a experiência com foco no momento presente, usando habilidades e qualidades da prática de meditação *mindfulness* para envolver os participantes em um treinamento intensivo com base na investigação experiencial^14^.

Alguns estudos avaliaram a viabilidade e os efeitos dos PBM tradicionais, como a Terapia Cognitiva Baseada em *Mindfulness* (TCBM) e a Redução de Estresse Baseada em *Mindfulness* (REBM), com resultados positivos na qualidade de vida e na autoeficácia dos participantes^15^. Elas são consideradas estratégias viáveis como tratamento complementar à Terapia Renal Substitutiva (TRS)^8,16^. No entanto, outros estudos relatam que os PBM convencionais, como REBM e TCBM, com sessões semanais de duas horas ou mais e longas práticas de *mindfulness* (45 minutos), podem diminuir a adesão de pessoas com insuficiência renal, especialmente aquelas em HD, devido ao estresse do tratamento em níveis físico, emocional e psicológico^17^, às demandas de tempo e aos custos de viagem^8,18^.

A viabilidade e os efeitos dos PBM adaptados às necessidades específicas de pessoas com alta demanda por serviços de saúde, como idosos^19^ e indivíduos em TRS^6,8,17,20,21,22,23,24^, têm melhorado. Reilly-Spong et al.^17^ adaptaram o modelo de REBM para um formato híbrido de teleconferência e reuniões presenciais para pessoas em TRS, demonstrando que esse modelo permitiu reduzir o tempo de deslocamento e os custos de viagem. Os participantes sugeriram realizar a intervenção durante a HD para ajudar no manejo da dor e da ansiedade que geralmente surgem durante as sessões. Entretanto, poucos estudos avaliaram a viabilidade de Intervenções Baseadas em *Mindfulness* (IBM) realizadas durante sessões de HD em adultos^8^. Esses estudos propõem intervenções com práticas breves de meditação *mindfulness* (5 a 30 minutos) com o suporte de áudios^20,22,23,24^ ou práticas guiadas utilizando óculos de realidade virtual (25 minutos) com recursos auditivos e visuais^21^ durante a HD. No entanto, nesses modelos, a prática compartilhada e a interação entre instrutor e participante são mínimas ou inexistentes. Esses elementos metodológicos são recomendados nas Diretrizes de Boas Práticas e Integridade em relação aos PBM, e as abordagens de terapia contextual são baseadas em atenção plena e aceitação^14,25^. Com o objetivo de ampliar a acessibilidade, reduzir o tempo e os custos de deslocamento e otimizar o tempo de tratamento, foi desenvolvido o Programa *Hemomindful*, um novo programa de manejo do estresse baseado em *mindfulness* durante as sessões de HD^6^.

Protocolos de pesquisa robustos e ensaios clínicos randomizados (ECR) para avaliar a viabilidade e os efeitos das IBM em pessoas com insuficiência renal são incipientes e escassos^7^. Embora estudos recentes nessa população indiquem que as IBM são viáveis e seguras, os aspectos do desenho do protocolo, a adesão às práticas e a satisfação dos participantes utilizando análise de métodos mistos precisam ser mais explorados. O presente estudo tem como objetivo descrever o protocolo de estudo de um ensaio clínico randomizado e avaliar a viabilidade e os efeitos preliminares do Programa *Hemomindful* realizado durante a hemodiálise.

## Método

### Desenho do Estudo

Os resultados apresentados são de um ensaio clínico randomizado controlado de métodos mistos para avaliar os efeitos do Programa *Hemomindful* combinado com o tratamento usual (TAU, por sua sigla em inglês) (grupo de intervenção - GI) em comparação ao TAU isolado (grupo controle - GC) em relação à dor e às condições de saúde em pessoas com insuficiência renal submetidas à HD, com um acompanhamento de até três meses.

Este estudo está registrado no site *Clinical Trials* (NCT 04610593) e foi aprovado pelo Comitê de Ética em Pesquisa do Hospital de Clínicas de Porto Alegre (HCPA) (CAAE: 40658214.6.0000.5336 GPPG / HCPA).

### Participantes

Foi selecionada uma amostra por conveniência. Os participantes foram elegíveis para o estudo se tivessem idade igual ou superior a 18 anos, estivessem em tratamento de HD há pelo menos três meses, falassem e entendessem a língua portuguesa, fossem alfabetizados, tivessem interesse em participar da pesquisa e obtivessem a pontuação mínima de corte (≥ 24) do mini exame do estado mental (MEEM)^26^. Os participantes foram excluídos se tivessem algum transtorno mental grave (diagnosticado pela equipe médica), estivessem clinicamente instáveis ou tivessem experiência prévia com meditação ou qualquer outra intervenção mente-corpo (por exemplo, ioga) nos últimos 12 meses.

### Intervenções

#### Tratamento usual na unidade de hemodiálise

Os cuidados habituais para pessoas com insuficiência renal que realizam HD foram oferecidos a todos os participantes por profissionais de diferentes áreas (medicina, enfermagem, nutrição, educação física, farmácia) que os assistem de acordo com o plano de tratamento individual.

#### Programa hemomindful

O programa é um PBM padronizado para manejo do estresse e promoção da qualidade de vida para pessoas com insuficiência renal submetidas à HD. A intervenção foi explicada em um manual e consistiu em oito sessões semanais de até 60 minutos, realizadas individualmente à beira do leito. Ela foi desenhada considerando as características e necessidades dessa população e com base no protocolo de REBM^27^, na Prevenção de Recaída Baseada em *Mindfulness* (PRBM)^12^, e no *Body in Mind Training* (BMT)^28^. O Programa *Hemomindful* inclui principalmente práticas de meditação *mindfulness*, além de estratégias de psicologia cognitivo-comportamental e educação em saúde para lidar com o estresse, a dor e os desafios relacionados à insuficiência renal e ao tratamento de HD.

O programa é dividido em três temas e práticas abrangentes com o objetivo de desenvolver habilidades de *mindfulness* de maneira gradual e progressiva, incentivando maior gentileza e aceitação das experiências ao longo do programa. A Tabela 1 apresenta os detalhes dos temas, objetivos, conteúdo didático e práticas para cada sessão.

**Tabela 1:** Programa *Hemomindful*

Tema	Sessões	Objetivo	Conteúdo educacional	Práticas e exercícios
Consciência do momento presente	1. Esteja presente	- Apresentar as bases do *Hemomindful*; - Apresentar o conceito de piloto automático e fazer um contraponto com o *mindfulness*; - Princípios do *mindfulness*: intenção, atenção e atitude (Modelo IAA)^36^; - Apresentar o *mindfulness* como uma forma de reconhecer padrões mentais; - Apresentar a pausa como uma forma de desacelerar e aumentar a consciência corporal.	- Apresentação do Programa; - Convite dos Três C’s: Coragem, Curiosidade, Compaixão; - *Mindfulness* versus piloto automático; - Modelo IAA^36^.	- Uva passa; - Pausa para o autocuidado com movimentos conscientes. *Tarefas de casa* - Pausa para autocuidado com movimentos conscientes; - Alimentação consciente.
	2. Habitando o corpo	- Apresentar as práticas de escaneamento corporal e movimentos conscientes; - Aumentar a atenção às reações físicas, cognitivas e emocionais aos gatilhos; - Apresentar os sistemas de regulação emocional: ameaça, recompensa e tranquilidade; - Demonstrar como essas reações nos levam a comportamentos automáticos e desviam nossa atenção do que realmente está acontecendo; - Apresentar movimentos conscientes como uma forma de autocuidado nesse processo automático.	- Triângulo da consciência: corpo, mente, emoções; - Desafios de práticas comuns; - Sistemas de regulação emocional.	- Escaneamento corporal; - Movimentos conscientes. *Tarefas de casa* - Escaneamento corporal e movimentos conscientes; - Pausas durante o dia; - Atividade diária com *mindfulness*.
	3. *Mindfulness* na vida cotidiana	- Apresentar as práticas de PARAR, sons e de caminhada consciente como possibilidades de integrar *mindfulness* na vida cotidiana; - Consciência da respiração como uma âncora sempre presente na experiência; - Aumentar a conscientização sobre o uso do PARAR para criar um espaço de resposta em vez de reação no dia a dia.	- Intenção: viver no momento presente; - Acrônimo PARAR (Parar, atentar, respirar, ampliar, responder); - Autorregulação da atenção.	- Escuta atenta; - Respiração; - PARAR para respirar. *Tarefas de casa* - Prática do *mindfulness* na respiração; - PARAR 3 vezes ao dia; - Mudança de hábitos: Caminhada consciente.
Aceitação e não reatividade	4. Acolhendo a dor e o desconforto	- Aumentar a consciência das sensações corporais, pensamentos e emoções que tendem a surgir em situações difíceis e estressantes; - Explorar reações primárias e secundárias a partir da experiência; - Praticar, permanecer e abrir-se para situações desafiadoras, como a experiência da dor e do desconforto, sem evitá-las ou fugir delas; - Aprender habilidades que ajudem a estar presente e a não ceder automaticamente à pressão e comportar-se de maneira disfuncional; - Explorar a descoberta de necessidades e atividades de autocuidado que realizam; - Introduzir a pausa com um gesto de auto passividade como prática de acolhimento e consciência compassiva, especialmente em situações difíceis.	- Aceitação versus luta ou fuga; - Experiência primária e secundária; - *Mindfulness* e experiência com dor e desconforto; - Autocompaixão.	- Surfar nas emoções; - PARAR em situações difíceis; - Listar atividades que ajudam a lidar com a dor e o desconforto; - Pausa autocompassiva *Tarefas de casa* - Surfar as dificuldades e pausa autocompassiva; - PARAR em situações difíceis; - Modificar algum hábito.
	5. Aceitação e ação hábil	- Cultivar uma atitude de curiosidade, bondade e não julgamento com as experiências neutras, agradáveis ou desagradáveis que surgirem; - Discutir o papel da aceitação no processo de mudança, especialmente em relação ao estresse e à dor; - Promover a experiência das qualidades de presença, enraizamento, dignidade, força e impermanência por meio de metáforas; - Explorar comportamentos habilidosos de autocuidado voltados para os valores da vida.	- Oração da Serenidade; - Poema “A casa de hóspedes”; - Sistema de calma.	- Sons, sensações, respiração, pensamentos e emoções; - Lista de ações para ativar o sistema de calma; - Meditação da montanha. *Tarefas de casa* - Sons, sensações, respiração, pensamentos e emoções; - Meditação da montanha; - Mudança de hábitos - Realizar uma atividade que ative o sistema de calma.
	6. Pensamentos não são fatos	- Reduzir o grau de identificação com nossos pensamentos, reconhecendo que não precisamos submeter-nos a eles ou controlá-los; - Discutir o ciclo de recaída de comportamentos reativos e compreender o papel dos pensamentos nesse processo; - Explorar a prática de movimentos atentos como uma âncora para a presença em momentos de fluxo mental acelerado.	- Hábitos mentais; - Mente cheia/vazia e mente atenta; - Lidar com “macacos mentais”.	- Pensamentos; - Movimentos conscientes; *Tarefas de casa* - Pensamentos e/ou movimentos conscientes; - PARAR 3 vezes ao dia - Observar os “macacos mentais”; - Atividade diária com *mindfulness.*
Mudança de hábitos e apoio social	7. Autocuidado e estilo de vida equilibrado	- Discutir a importância de um estilo de vida equilibrado e cuidar de si mesmo para reduzir a vulnerabilidade à recaída de comportamentos reativos; - Introduzir a prática da bondade amorosa como mais uma prática de consciência e atitude compassiva em relação a si mesmo e aos outros; - Discutir a prática regular do *mindfulness* como forma de manter o equilíbrio.	- Equilíbrio entre estilo de vida e compaixão para uma vida saudável e plena; - Consciência de atividades, pessoas e situações associadas a desconforto e emoções desafiadoras, prazer e energia e neutralidade.	- Bondade amorosa; - Listar atividades exaustivas, neutras e energizantes; - PARAR na respiração. *Tarefas de casa* - Bondade amorosa e outros de sua escolha; - Atividade de energização; - Relato, objeto, desenho, música, etc... que represente o que você aprendeu, vivenciou ou teve valor no Programa *Hemomindful.*
	8. Apoio social e prática contínua	- Destacar a importância das redes de apoio e a importância do equilíbrio no estilo de vida, cuidando de si mesmo para reduzir o estresse; - Discutir o uso regular do *mindfulness* como um meio de manter o equilíbrio; - Refletir sobre o que foi aprendido no curso, os motivos e as estratégias para prosseguir com a prática.	- Poema “Paradoxo”; - Revisão de conteúdos, práticas e intenções; - Facilitadores e barreiras à prática contínua; - Valores e ações comprometidos.	- Escaneamento corporal; - Conclusão da meditação.

Foram trabalhadas quatro técnicas principais de meditação *mindfulness* formal: respiração consciente, escaneamento corporal, movimentos conscientes e prática compassiva, bem como práticas curtas para serem utilizadas informalmente na vida cotidiana, seja para sair do piloto automático ou para lidar com situações desafiadoras, como a dor. As práticas breves incluem: Pausa para autocuidado, PARAR (Parar, atentar, respirar, ampliar, responder) e Pausa para autocompaixão.

Cada sessão apresenta momentos diferentes: Verificação (como o participante está naquele momento); prática de *mindfulness*; investigação da experiência; psicoeducação; tarefas de casa; e verificação de encerramento. O conceito e a habilidade de *mindfulness* são desenvolvidos a partir da experiência com exercícios e práticas de áudio de 5 a 20 minutos pelo instrutor, nas quais o participante e o instrutor praticam juntos com o suporte de fones de ouvido compartilhados e diferentes práticas guiadas. Em cada sessão, materiais informativos sobre o tema da sessão, exercícios e um diário foram distribuídos aos participantes para que descrevessem suas experiências com as práticas durante a semana.

Além disso, os participantes foram incentivados a realizar práticas formais e informais em casa e nos outros dias, pelo menos uma vez ao dia. Para aprofundar sua prática, eles receberam áudios de práticas guiadas gravados pelo instrutor em CD ou em formato digital. Ao final da intervenção, os materiais informativos utilizados em cada sessão foram compilados em um manual para apoiar a prática contínua.

O Programa *Hemomindful* foi conduzido por um instrutor certificado em *mindfulness*, treinado nos protocolos de PRBM e BMT, além de prática meditativa consolidada e com mais de dez anos de experiência. O instrutor fazia parte da equipe multiprofissional da unidade de HD onde o estudo foi realizado.

Foram feitas anotações em diários de campo e todas as sessões foram gravadas em áudio durante a intervenção para documentar o recebimento do tratamento pelos participantes e quaisquer desvios dos checklists da intervenção.

### Procedimentos

A triagem envolveu a verificação da lista de indivíduos atendidos na Unidade de HD e a identificação daqueles que preenchiam os critérios de elegibilidade utilizando informações dos prontuários médicos eletrônicos. Em seguida, o coordenador do projeto explicou detalhadamente a pesquisa a cada participante em potencial, informando sobre a natureza voluntária da participação e que eles poderiam retirar-se do estudo a qualquer momento, sem que isso afetasse seu tratamento. Aqueles que concordaram em participar assinaram o termo de consentimento livre e esclarecido. Uma triagem final e outras fases de coleta de dados foram realizadas presencialmente por entrevistadores treinados. Após a coleta de dados basais, os participantes do grupo intervenção (GI) receberam informações sobre o Programa *Hemomindful*, enquanto os participantes do grupo controle (GC) permaneceram na rotina de TAU, sem receber instruções adicionais, e foram lembrados de que, após a conclusão do estudo, poderiam realizar o programa caso desejassem. Os participantes não eram cegos quanto ao status do grupo. O tamanho da amostra foi estimado com base em pesquisas anteriores e em diferenças entre os grupos em um nível de significância de 5%, com poder de 85% e tamanho de efeito d de Cohen = 1,0. Isso exigiu o registro de 30 a 40 participantes (15 a 20 participantes por grupo). A randomização foi realizada utilizando o método de alocação aleatória estratificada 1:1. Durante o período de recrutamento (abril a junho de 2019), 32 participantes foram inscritos, com 16 indivíduos randomizados para o programa *Hemomindful* (GI) e 16 indivíduos para o TAU (GC) (Figura 1). A sequência de randomização foi criada com o ambiente estatístico *R* usando o pacote *minDiff* ^29^ por um pesquisador que não esteve envolvido nas avaliações nem na seleção dos participantes.

**Figura 1:** Fluxograma do estudo.

Nota – TAU: Tratamento usual.

Os resultados relacionados aos efeitos do programa incluíram medidas quantitativas e qualitativas autorrelatadas e níveis de biomarcadores inflamatórios medidos no início do estudo, após 8 semanas e após 3 meses da intervenção (janeiro a maio de 2020). A dor, avaliada pela escala *Profile of Chronic Pain: Screen - PCP:S* ^30^, foi o desfecho primário do estudo. Este trabalho se concentrará no desenho do estudo, nos desfechos de viabilidade e nos efeitos percebidos pelos participantes do programa *Hemomindful*, enquanto os desfechos completos de eficácia do programa, comparando GC e GI, serão relatados em outro momento.

### Medidas de Desfecho

O perfil dos participantes foi avaliado por meio de um questionário com informações sociodemográficas e clínicas, desenvolvido pela equipe de pesquisa para obter informações como idade, raça, escolaridade, nível econômico dos participantes segundo os critérios da Associação Brasileira de Empresas de Pesquisa (ABEP)^31^ comorbidades, tempo de tratamento e meditação prévia.

O MEEM foi utilizado para rastrear déficits cognitivos^26^. O MEEM é composto por questões que se correlacionam em cinco dimensões: concentração, linguagem/praxia, orientação, memória e atenção, com pontuação máxima de 30 pontos. O ponto de corte 23/24 é o mais comumente utilizado. Esse instrumento foi aplicado e validado para o contexto brasileiro, demonstrando alta sensibilidade e especificidade na detecção de comprometimento cognitivo^26^.

A viabilidade foi avaliada pelo número de participantes que completaram o protocolo, adesão ao Programa *Hemomindful*, implementação, segurança e satisfação com o tratamento. A retenção ao protocolo do estudo foi avaliada por meio dos dados primários de todos os participantes no início do estudo.

A adesão ao Programa *Hemomindful* foi mensurada por meio dos registros semanais da equipe de pesquisa nos diários de campo sobre a participação em cada sessão e a frequência da prática formal e informal de *mindfulness* realizada em domicílio, relatada pelos participantes (número de dias por semana). A implementação e a segurança foram avaliadas com base em registros de diários de campo sobre eventos adversos observados pela equipe de pesquisa.

A satisfação com o tratamento e a experiência dos participantes foram avaliadas imediatamente após a conclusão da intervenção (8 semanas após a randomização) por meio de uma entrevista individual qualitativa semiestruturada utilizando um questionário para reflexões sobre o programa *Hemomindful*, adaptado de Bowen e colaboradores^12^. O questionário continha questões fechadas (respostas em escala Likert de 0 a 10; “10 = muito provável/muito importante”) e questões abertas abordando tópicos como o grau de importância do programa, mudanças percebidas, a intervenção durante as sessões de HD, a continuidade das práticas de *mindfulness* após a intervenção e sugestões para aprimorar a intervenção para uso futuro. As entrevistas foram gravadas digitalmente, anonimizadas e transcritas na íntegra para análise. A duração das entrevistas variou de 15 a 30 minutos.

### Análise dos Dados

Para dados quantitativos, foi realizada uma análise estatística descritiva. As variáveis contínuas são expressas como média ± desvio padrão. As variáveis categóricas são expressas em frequência e proporção. As comparações foram avaliadas pelo teste χ^2^ de Pearson ou pelo teste t de Student. Todas as análises numéricas foram realizadas no SPSS versão 23. Os dados qualitativos sobre a satisfação com o tratamento e a experiência dos participantes do Programa *Hemomindful* foram transcritos literalmente e utilizou-se o método de análise textual discursiva, que consiste em três etapas: unitarização, categorização e análise das categorias^32^. A análise foi realizada por dois codificadores independentes. A codificação foi baseada em uma abordagem semântica na qual os códigos são derivados do significado explícito dos dados. Em seguida, eles codificaram de forma independente os dados sobre os principais temas e categorias temáticas. A categorização dos dados foi concluída por meio de um processo interativo e de uma abordagem de consenso para discordâncias.

A análise descritiva quantitativa das características da amostra foi realizada para os grupos GC e GI, enquanto a análise qualitativa foi realizada apenas para o grupo GI.

## Resultados

### Características Basais

Dos 67 voluntários potenciais, 32 foram selecionados (28 eram inelegíveis e 7 se recusaram a participar) (Figura 1). A média de idade foi de 55 anos (mín. 24, máx. 84, DP = 15,6), a maioria era composta por mulheres (n = 19; 59,4%), os pretos e pardos (de acordo com a cor da pele autorrelatada) representaram 50% da amostra (n = 16) e a maioria vivia com membros da família (n = 29; 90,6%). Dezenove tinham ensino fundamental ou superior (59,4%), 15,6% (n = 5) exerciam atividade remunerada e a maioria dos participantes pertencia à classe econômica C (n = 15; 46,8%). A duração da terapia de HD, em média, foi de 6,28 anos (mínimo 0,3; máximo 20,3; DP = 5,67) e os participantes apresentaram uma média de 3,94 comorbidades (DP = 2,32) associadas à insuficiência renal, sendo a dor a mais prevalente (n = 19; 59,37%). Os grupos foram semelhantes em termos de características sociodemográficas e clínicas (Tabela 2).

**Tabela 2:** Características basais dos participantes randomizados para o Programa *Hemomindful* ou para Tratamento Usual (TAU) (n = 32)

Variáveis	*Hemomindful* (n = 16) Média ± DP	TAU (n = 16) Média ± DP	
	N (%)	N (%)	*Estatísticas*
**Idade** (anos)	52,50 ± 16,16	58,25 ± 14,9	*t* ^a^ = –1,04; *p* = ,304
**Sexo**			*χ* ^2b^ = 1,16; *p* = ,280
Homem	8 (50)	5 (31,2)	
Mulher	8 (50)	11 (68,8)	
**Raça**			*χ* ^2b^ = 1,06; *p* = ,301
Branca	7 (43,8)	8 (50,0)	
Preta	6 (37,5)	6 (37,5)	
Parda	2 (12,5)	2 (12,5)	
Amarela	1 (6,2)	0 (0,0)	
**Mora com**			*χ* ^2b^ = ,36; *p* = ,544
Sozinho(a)	2 (12,5)	1 (6,2)	
Com a família	14 (87,5)	15 (93,8)	
**Educação**			*χ* ^2b^ = ,58; *p* = ,443
Ensino fundamental incompleto	7 (43,8)	6 (37,5)	
Ensino fundamental completo	2 (12,5)	3 (18,8)	
Ensino médio incompleto ou completo	5 (31,2)	4 (25,0)	
Ensino superior incompleto ou completo	2 (12,5)	3 (18,7)	
**Situação de Emprego**			*χ* ^2b^ = ,23; *p* = ,238
Trabalho remunerado	3 (18,8)	2 (12,5)	
Aposentadoria	13 (81,2)	14 (87,5)	
**Nível econômico**			*χ* ^2b^ = ,73; *p* = ,391
A	1 (6,2)	1 (6,3)	
B1- B2	5 (31,3)	7 (43,7)	
C1 – C2	8 (50,0)	7 (43,7)	
D – E	2 (12,5)	1 (6,3)	
**Tratamento**			
Anos em hemodiálise	6,85 ± 6,22	5,72 ± 5,2	*t* ^a^ = ,55; *p* = ,581
Comorbidades	4,69 ± 2,58	3,19 ± 1,83	*t* ^a^ = 1,89; *p* = ,068
Uso de medicamentos psicotrópicos	9 (56,3)	5 (31,3)	*χ* ^2b^ = 3,27; *p* = ,071

Nota – DP: Desvio padrão; Dados apresentados como média ± DP ou n (%). TAU: Tratamento Usual; *Hemomindful*: Programa *Hemomindful*.

*^a^Teste *t* de Student.

^b^Teste qui-quadrado de Pearson.

### Retenção no Protocolo e Dados Basais

Nenhum dos participantes (n = 32) abandonou o protocolo (retenção de 100%). Os dados basais foram coletados de todos os participantes no início do estudo. No grupo GI, houve quatro desistências ao longo das 8 semanas (25%) devido a “cansaço” e “falta de motivação para praticar” (n = 1), transferência para outra unidade de tratamento (n = 1), transplante (n = 1) e óbito (n = 1). No grupo GC, um voluntário (6,25%) foi perdido devido a transplante renal. A taxa geral de retenção ao protocolo do estudo foi de 84,38%.

### Adesão, Implementação e Segurança do Programa *Hemomindful*

Dos participantes do grupo GI (n = 16), 93,75% (n = 15) realizaram quatro ou mais sessões do Programa *Hemomindful* e 75% (n = 12) completaram as oito sessões programadas. Algumas sessões precisaram ser reagendadas porque o participante não se encontrava bem no horário agendado (por exemplo, náusea, vômito, complicações no acesso à HD) e foram realizadas em outro dia de HD planejado para o participante.

Durante o estudo, todos os participantes forneceram dados sobre a prática de *mindfulness* em casa, sendo que 12 forneceram dados completos para as semanas 1–8 (período de intervenção) e quatro participantes forneceram dados parciais, que foram utilizados para estimar a média de dias de prática domiciliar por semana. Os participantes que concluíram as oito sessões do Programa *Hemomindful* (n = 12) relataram uma prática formal de *mindfulness* por, em média, 1,88 (DP = 1,97) dias e por um total de 13,17 minutos (mín. 1, máx. 49; DP = 13,75) e uma prática semanal curta ou informal de 3,79 (DP = 2,15). O programa teve duração de 26,50 dias (mín. 7, máx. 49; DP = 15,07) (dias entre a primeira e a oitava sessões). O envolvimento médio semanal com práticas de *mindfulness* em casa foi diferente de acordo com a semana avaliada. As práticas formais foram mais frequentes nas semanas 3 e 6 e as práticas informais nas semanas 1 e 3 (Tabela 3).

**Tabela 3:** Frequência média (em dias por semana) de práticas formais e informais de *mindfulness* em casa e ao longo do período

Semana	N	Práticas formais		Práticas informais	
		Média	DP	Média	DP
Semana 1	16	1,94	2,26	5,0	3,09
Semana 2	16	0,88	1,92	2,5	3,20
Semana 3	15	2,0	1,89	4,73	3,01
Semana 4	14	1,87	2,03	3,87	3,20
Semana 5	13	1,62	2,14	2,23	3,24
Semana 6	12	2,38	2,39	3,46	3,25
Semana 7	12	1,33	2,06	2,0	3,13
Todo o período	12	13,17	13,75	26,50	15,07
Média semanal total	12	1,88	1,97	3,79	2,15

Nota – DP: Desvio padrão.

Os participantes receberam 13 áudios de práticas de *mindfulness*, com duração entre 3’27” e 16’50”, totalizando 147 minutos de prática guiada. As principais práticas formais trabalhadas foram pausas para autocuidado (n = 12), respiração (n = 10) e meditação da montanha (n = 10). A prática informal mais prevalente foi a pausa ou PARAR (Parar, atentar, respirar, ampliar, responder) mencionada por todos os participantes (n = 16). A prática de *mindfulness* durante a alimentação e os movimentos conscientes foram mencionados 25 e 8 vezes, respectivamente, no decorrer do programa. Os participantes também relataram realizar práticas informais de *mindfulness* ao brincar com seus filhos e netos, durante o banho, ao escovar os dentes e ao cuidar do jardim. Além disso, relataram mudanças em seus hábitos, como assistir menos televisão, diminuir o uso de celulares, observar a natureza, pintar e preocupar-se mais com a aparência, entre outros.

Durante o estudo, nenhum participante relatou eventos adversos graves ou inesperados. Alguns participantes notaram efeitos colaterais leves e/ou moderados durante a intervenção, como aumento da percepção da dor em práticas de movimento consciente ou quando convidados a investigar a experiência da dor (n = 3) e da ansiedade (n = 3) durante a prática.

### Satisfação e Experiência com o Tratamento

Os participantes que concluíram o Programa *Hemomindful* (n = 12, 75%) classificaram-no como “muito importante”, com uma pontuação média de 8,58 (DP = 2,06). Quando questionados sobre o motivo da pontuação atribuída, eles mencionaram que o programa foi importante por diferentes razões, como: “reduzir o estresse”, “aprender coisas novas”, “benefício espiritual”, “tranquilidade”, “força de vontade”, “mais ânimo”, “energia”, “disposição”, “aprender”, “mudar para melhor”, “conhecer-se melhor”, “aproveitar as coisas boas”. Entre os participantes que atribuíram valores mais baixos à importância do programa (n = 2), um não soube responder o motivo, enquanto o outro disse que o programa não correspondeu às suas expectativas.

Em relação ao grau de interesse em *mindfulness* ao final da intervenção, os participantes apresentaram uma pontuação média de 8,5 (DP = 2,15) em uma escala Likert de 0 a 10. Quanto à probabilidade de continuarem a realizar práticas formais de *mindfulness* (por exemplo, *mindfulness* de respiração, escaneamento corporal, movimentos conscientes, entre outros) e práticas informais (PARAR, alimen­tação e caminhada conscientes, entre outros), eles indicaram uma pontuação média de 6,67 (DP = 2,93) e 8,5 (DP = 2,31), respectivamente.

A partir das respostas às questões qualitativas abertas sobre sua experiência com o Programa *Hemomindful*, emergiram dois temas amplos: a percepção dos participantes sobre as mudanças percebidas e os comentários sobre a estrutura do programa, subdivididos em três categorias cada, com citações representativas apresentadas na Tabela 4.

**Tabela 4:** Análise qualitativa da satisfação e da experiência com o Programa *Hemomindful*

Categorias	Discursos representativos dos participantes
**Tema 1. Mudanças percebidas**	
*Mindfulness* na vida cotidiana.	*"Por exemplo, tomar banho. Eu tento ser mais rápido no banho, porque presto muita atenção para não cair, preciso prestar muita atenção ao me vestir. Tento prestar atenção no que estou fazendo para não deixar minha mente vazia e não fazer as coisas rapidamente para não ser um problema". (P2)* *"A questão dos hábitos alimentares mudou, antes eu não comia frutas agora eu já como, então isso mudou muito na minha saúde, porque eu aprendi a comer melhor, a ter mais paciência também para comer, porque antes eu tentava fazer tudo correndo, agora não, eu tento fazer tudo com calma". (P12)* *"Ah... como eu posso te dizer... é... postura. Sim, a postura mudou, observação". (P1)* *"Isso mudou alguma coisa durante meu dia ou durante o período em que vou fazer as orações, adapto o que aprendi e aumento ainda mais minha prática". (P2)* *"Mudou um pouco, muda, tudo muda, a memória de tudo o que você viu, as respostas, as coisas que você ouviu, tudo muda". (P5)* *"Meu comportamento, pensamento, mudou muito. Em relação à família, filha, meu comportamento mudou para melhor". (P6)*
Não reatividade.	*"Ah, o autocontrole dos meus desejos, desejo prático, água, comida, coisas assim. Eu penso bem, se eu realmente preciso, se não, posso esperar um pouco mais para beber, isso eu tenho feito direito. Estou tentando, me segurando, tentando lidar com a sede". (P8)* *"Eu achei mais valioso tentar dominar a decisão, aquela decisão explosiva, aquela decisão do momento, que eu achava legal, era algo que eu estava tentando fazer, dar um passo atrás e respirar fundo". (P8)* *"Aprendi a me controlar, coisa que antes eu não me controlava, para mim foi muito bom ter feito isso, porque antes eu me irritava muito, e agora aprendi isso, a me controlar, tenho que esperar um pouco, depois também penso para responder, eu não pensava nisso antes". (P12)* *"(...)minha maneira de agir mudou mais, minha nova maneira é a seguinte, primeiro eu penso e depois faço, no começo eu não pensava mais, eu ia lá e fazia e dava tudo errado, melhorou muito a maneira com que eu agia com o corpo hein". (P4)* *"Sim, quando eu estou muito agitado, eu paro, penso, respiro, certo? e volto com calma". (P1)* *"Isso muda a maneira de pensar, a maneira de agir, eu faço minhas coisas com mais disposição". (P10)*
Lidando com a dor e o desconforto.	*"Achei que foi uma experiência de aprendizado para nós, sabe, é uma autoajuda (...) se hoje eu não estiver muito bem e não quiser estar nessa situação, então posso tentar melhorar, e isso realmente ajuda". (P10)* *"As coisas ruins passam, como se nem... vai embora, vai embora, isso também me estimulou muito... é como pensar que não vai dar certo, não, vai dar. Eu tinha pensamentos ruins, eu era negativo, a prática me ensinou muito". (P11)* *"Ah, porque era possível evitar o estresse, você vê que está errado... (silêncio)". (P1)* *"O mais legal, porque coisas que aconteciam lá atrás eu não conseguia lidar com elas e, depois que comecei a fazer isso, consegui fazer com que tudo mudasse para melhor". (P6)* *"Eu aprendi que nós podemos (...) sair dessa situação”. “Porque, às vezes, quando estou realmente mal, volto e dou uma olhada no livro, recorro ao livro de práticas". (P10)* *"Física eu acho que não, mas o emocional tem um bom significado. Não fico mais tão nervoso, não penso, não me importo muito". (P3)* *"Na verdade, eu consegui lidar com a questão emocional. Muitas vezes, eu vinha para cá agitado por causa dos problemas em casa e depois o estudo tocava meu corpo e minha mente. Quantas vezes eu cheguei aqui mal, até pensando em desistir de tudo, mas eu ia, ia, ia, até o final". (P4)* *"(...)Antes eu não sabia como lidar com meu desconforto e hoje eu já sei. Penso nas práticas que fiz, penso nelas, respiro fundo e o desconforto passa". (P6)* *"Não. Porque se eu pensar no braço, ele dói! Daí dói!". (P7)* *"Eu percebi que, como relaxo muito, consigo relaxar". (P2)* *"Eu tento entender melhor onde está a dor, tento, como é... entrar na dor para ver o que posso fazer. É diferente, antes eu sentia dor, tinha sintomas e tomava remédios, às vezes ficava irritado. Quando não temos o controle de algo, é muito frustrante, é muito frustrante tentar e falhar, essa sensação muito ruim de impotência". (P8)*
	*"O desconforto é mais emocional, com essa máquina grande, como um incômodo. Mas pouco mudou com as práticas". (P8)* *"(...)Minha vida agora se resume a uma máquina, a não ir para Capão (praia), eu poderia muito bem ir para lá nos fins de semana, ver meus filhos lá. Eu não posso, você tem que vir aqui, às vezes isso irrita, então é uma forma de lidar com a dor, a dor emocional, dor física... é muito estranho". (P2)* *"Para mim, continua a mesma coisa". (P9)* *"Sim, eu percebi uma mudança, porque antes eu reclamava das minhas dores e agora não reclamo mais, agora posso enfrentar a dor sem reclamar e ela diminuiu bastante". (P12)*
**Tema 2. Estrutura do programa *Hemomindful***	
Melhor utilização do tempo de HD.	*"Ótimo, 10, porque as horas passam rapidamente e você não fica agitado aqui". (P1)* *"Eu achei legal, não sei, porque isso ajuda muito a não ficar ansioso por querer sair daqui rápido". (P3)* *"Eu achei muito legal, porque é algo que está ajudando, me ajudou muito, achei muito legal". (P6)* *"Bom, porque pelo menos você fica aqui por 4 horas, às vezes eu me sinto bem, às vezes eu me sinto mal, mas você está lá querendo que tudo saia bem, aí vem aquelas palavras legais do professor, você gosta disso". (P2)* *"Eu tenho mais paciência agora para esperar todas essas horas, o que costumava me irritar muito, mas agora não". (P12)* *"Muito bom, porque é um tempo bem gasto". (P8)*
Materiais, práticas e equipe.	*“Eu aprendi tudo, apesar da memória, as coisas que ela diz e o toque do sino”. (P5)* *“Bom, aquele sino era muito bom, aqueles fones de ouvido, ela falando...”. (P2)* *“Tudo era importante, todas as palestras (...) tem aquela sobre a montanha, o macaco, a pedra.” (P5)* *“Porque, às vezes, quando estou muito para baixo, eu dou uma olhada no livro, recorro ao livro de práticas”. (P10)* *“Eu acho que as práticas poderiam ser mais objetivas e com menos tempo de prática (...) e o formato delas não precisaria ser tão igual, poderia ser mais diversificado, porque sempre começa com a mesma coisa e termina com a mesma coisa, que a prática inteira não levasse mais de uma hora”. (P8)* *“Eles são bons, foram bem aproveitados, ajudam, dão atenção, as palavras, conversam com a gente, são pessoas diferentes é bom, muito bom”. (P2)* *“Que foi muito bom, a professora foi muito legal, ela tem toda a paciência com você, e ela se sente tão bem em fazer isso, ela nos fez sentir tão bem, ela quer que a gente fique bem também, eu não sei como me expressar...”. (P1)* *“A maneira de ensinar e o que aprendi eu coloquei na minha cabeça e no meu coração e ainda sigo em frente, ainda está dentro de mim, não saiu, não foi embora, assim como naquele dia em que você me fez uma pergunta e eu entendi o que era". (P4)* *“Todos os oito programas foram bons". (P4)* *“Eu uso o livrinho, sou um pouco preguiçoso, sabe como é". (P10)* *“Não teve nada que me atrapalhasse, pelo contrário, fui super ajudado". (P12)*
Continuidade do programa.	*“Foi muito bom e outra coisa, a prática desse trabalho que começou a ser feita e não pode parar, porque traz muito benefício para nós, e a gente faz isso como lavar a mente e não deixar que ela se sobrecarregue, temos que manter para relaxar". (P2)* *“Gostaria que todo mundo parasse, pensasse e fizesse isso, porque é muito bom, ajuda muito”. (P12)* *“Eu acho que tenho que continuar, fazer com outras pessoas, assim como me ajudou, vai ajudar muitas outras pessoas que estão realmente precisando, continue fazendo esse projeto que vai ajudar muitas pessoas". (P6)*

Nota – P: Participante.

Ao analisar as mudanças percebidas, surgiram três categorias: *mindfulness* nas atividades diárias, não reatividade e manejo da dor e do desconforto. Os participantes relataram que o programa contribuiu para um aumento do *mindfulness* nas atividades diárias, ajudando-os a estar mais presentes e conscientes na realização de tarefas cotidianas, como tomar banho, comer, caminhar e vestir-se. Além disso, notaram mudanças nas relações interpessoais e na forma como se relacionavam com as demandas do dia a dia, adotando uma postura de maior observação e tranquilidade. Quanto à não reatividade, muitos participantes perceberam uma mudança ao se relacionarem com experiências internas e externas, especialmente em relação a experiências desagradáveis. Os relatos expressaram como eles aprenderam a “recuar e respirar fundo” para fazer escolhas conscientes, em vez de simplesmente reagirem automaticamente à experiência. Alguns participantes comentaram sobre o uso de práticas de *mindfulness* para lidar com os desafios da insuficiência renal e da TRS, como o tempo de HD, a restrição hídrica e as alterações na dieta impostas pelo tratamento. Em relação ao manejo da dor e do desconforto, os participantes observaram mudanças ao lidar com esses fatores de estresse. Eles relataram que a prática de *mindfulness* os auxiliou a lidar com sensações físicas e pensamentos e emoções desagradáveis, além de fazer escolhas que ajudaram em seu autocuidado e na regulação de suas emoções. Foram mencionadas atitudes como reconhecer e explorar a dor sem tentar controlá-la, bem como pensamentos e emoções de “deixar passar” e o uso de recursos como o PARAR para respirar e realizar movimentos conscientes. Houve relatos de redução da dor e mudanças positivas em nível emocional. Uma participante relatou que sua dor aumentou ao observar as sensações de seu corpo, enquanto outra disse que não percebeu nenhuma alteração relacionada à dor.

No tema da estrutura do programa, surgiram as seguintes categorias: melhor aproveitamento do tempo de HD; materiais, práticas e equipe; e continuidade do programa. A melhor utilização do tempo de HD foi mencionada por 50% (n = 6) dos participantes que concluíram a intervenção. Eles consideraram que, além de sentirem o tempo passar mais rapidamente, poderiam usar esse tempo para aprender a praticar *mindfulness* e lidar com a ansiedade e a irritação que surgem durante a HD. Em relação aos materiais, os participantes apreciaram o uso de alguns itens durante a intervenção, como o sino, os fones de ouvido e o manual do praticante. A maioria dos participantes ficou satisfeita (ou “muito satisfeita”) com as práticas de *mindfulness*, com um participante sugerindo que as práticas formais poderiam ser mais objetivas e diversificadas. A maioria também demonstrou satisfação com o conteúdo e a duração das sessões do Programa *Hemomindful*, com somente um participante sugerindo que as sessões poderiam durar menos de uma hora. A maior parte dos participantes relatou que a presença e a atitude da equipe de pesquisa, bem como a investigação das experiências por parte do instrutor, possibilitaram uma boa interação e troca de experiências. A maioria dos participantes relatou que daria (ou provavelmente daria) continuidade ao programa por considerá-lo útil e por ter proporcionado oportunidades importantes de aprendizado.

## Discussão

Nosso estudo demonstra que o Programa *Hemomindful* apresentou indicadores positivos de viabilidade. A taxa de retenção no Programa *Hemomindful* foi elevada em comparação aos resultados de estudos anteriores com pessoas com insuficiência renal^17,20,21,23,24^, com 93,75% dos participantes tendo realizado quatro ou mais sessões e 75% concluindo as oito sessões do programa. Thomas et al.^24^ apresentaram uma taxa de retenção de 71% em uma IBM com práticas breves de *mindfulness* durante a HD em comparação com o TAU isolado. No estudo de viabilidade de uma REBM^17^ adaptada, realizado fora do contexto do tratamento de HD, com duas sessões presenciais e seis sessões por teleconferência, 84% dos participantes realizaram três ou mais sessões e somente 36% concluíram o programa. Outros estudos que investigaram a viabilidade e os efeitos das IBM na redução do estresse em pessoas com insuficiência renal, realizados fora do contexto utilizando protocolos tradicionais de grupo, como a TCBM^15^ e a REBM^33^, não relataram claramente as taxas de adesão dos participantes. As desistências em nosso estudo ocorreram por motivos semelhantes aos de estudos anteriores: cansaço, falta de interesse em realizar o programa, transferência para outra unidade, transplante, deterioração da saúde e óbito^15,17,24^.

Os participantes relataram a prática formal de *mindfulness* em casa, em média, um ou dois dias por semana. Somente um estudo sobre IBM para pessoas com doença renal em estágio terminal investigou seu envolvimento com práticas formais de *mindfulness* em casa, e os participantes relataram praticar três vezes por semana, em média^24^. Ribeiro et al.^19^ avaliaram a adesão de idosos (50–80 anos) com múltiplas comorbidades à prática de *mindfulness* durante e após uma intervenção de 6 semanas realizada individualmente e não encontraram associação entre a duração do engajamento e alterações no estado psicológico e na qualidade de vida. Um ECR que avaliou os efeitos da TCBM na prevenção da recaída de depressão mostrou uma associação inversa entre a duração da prática formal de *mindfulness* realizada em casa e a probabilidade de recidiva da depressão^34^.

Práticas informais de *mindfulness*, 4 ou 5 dias por semana, foram relatadas por todos os participantes. Outros estudos também demonstraram maior adesão às práticas informais de *mindfulness* ^33,34,35^. Pesquisas realizadas com pessoas que praticam *mindfulness* há um ano ou mais mostraram que a frequência da prática informal é mais importante para aumentar o bem-estar psicológico e a flexibilidade do que a prática formal^34^.

A satisfação com o Programa *Hemomindful* foi evidenciada por meio de resultados quantitativos e qualitativos, com os participantes atribuindo um grau de importância (8,5) semelhante ao de outro estudo realizado durante as sessões de HD (8,3)^24^ e superior ao de um estudo com TCBM adaptada para teleconferência (8,0)^17^. Nossos resultados indicam que a participação no Programa *Hemomindful* contribuiu para a ampliação das práticas de *mindfulness* dos participantes para além do contexto da HD, especialmente na realização de atividades da vida diária, como comer, vestir-se, caminhar, conversar com amigos e familiares, entre outras. Nossos resultados apontam para uma redução da reatividade a situações cotidianas relacionadas tanto a fatores associados ao tratamento da insuficiência renal, como restrições alimentares e hídricas ou uso de medicamentos, quanto a diversas situações pessoais e familiares. O modo habitual do ser humano é a *“maneira de fazer”*, de solucionar problemas com o objetivo de reduzir a distância entre onde estamos e onde gostaríamos de estar. Esse modo é eficaz para resolver problemas externos, mas, quando se trata de resolver problemas internos, ele apenas cria mais dificuldades. Isso ocorre porque, ao tentar buscar as causas e os motivos pelos quais as coisas não estão como gostaríamos, frequentemente surgem pensamentos de análise, julgamento e comparação que podem gerar culpa, ansiedade e estresse^12,13^. A capacidade do *mindfulness* de perceber conscientemente as experiências internas e externas de forma atenta, gentil, curiosa e sem julgamentos é o oposto do modo automático e reativo^12,36^.

Um melhor manejo da dor e do desconforto foi uma das mudanças percebidas pelos participantes. *Mindfulness* é um construto importante no campo da dor crônica em adultos, pois enfatiza uma atitude de não julgamento para com a experiência do momento presente, observando cuidadosamente - em vez de reagir automaticamente - às sensações físicas, emoções e pensamentos que estão presentes^27,37^. Os PBM são considerados um tratamento complementar promissor para pessoas que sofrem de dores, como cefaleia^38^, dor nas costas^35^ e fibromialgia^39^. Estudos sugerem que a prática regular de *mindfulness* pode levar a mudanças na aceitação da dor e na percepção de sua intensidade^39,40^.

Poucos estudos avaliaram a viabilidade das IBM no contexto da HD, as quais também se mostraram viáveis^21,24^. Estudos investigam a viabilidade de uma intervenção adaptada do Programa *Hemomindful* que siga as recomendações internacionais sobre PBM para pesquisa clínica e educacional, com a prática de *mindfulness* como elemento central, com bases conceituais sólidas e suporte psicoeducacional adequado^14,25^. A análise qualitativa destacou diferentes aspectos do programa, como o papel do instrutor e da equipe de pesquisa, a maneira de ensinar, a forma como os instrutores fizeram perguntas aos participantes sobre suas experiências e lhes deram a oportunidade de conversar com outras pessoas. A importância da experiência do processo de investigação para o desenvolvimento de habilidades de *mindfulness* é descrita em outros estudos como um elemento essencial na aprendizagem e no processo de mudança^12,14^.

O uso de diferentes materiais educativos durante a intervenção também foi valorizado pelos participantes. Esses recursos são utilizados para aumentar o foco durante a prática e facilitar o aprendizado^6,34^, e a decisão de utilizá-los foi baseada nas dificuldades relatadas em outros estudos no contexto da HD^24^, bem como nas recomendações de estudos com pessoas com insuficiência renal fora do contexto da HD^17,18^.

Outro aspecto importante é que o Programa *Hemomindful* foi conduzido por um instrutor pertencente à equipe de diálise, o que pode ter contribuído para a adesão à intervenção, uma vez que os PBM desenvolvidos por profissionais da equipe de cuidados, treinados e certificados, normalmente apresentam melhores resultados de adesão^40^.

### Limitações e Direções Futuras

Nosso estudo apresenta algumas limitações que devem ser observadas. Primeiramente, foi utilizada uma amostra de conveniência de pacientes de uma única unidade de HD, o que pode ter introduzido algum viés nos resultados. Em segundo lugar, o Programa *Hemomindful* é um programa multifacetado, composto por diferentes práticas de *mindfulness* e conteúdos e técnicas psicoeducacionais, o que dificulta o discernimento da importância relativa de cada elemento do programa. Estudos futuros devem, portanto, realizar ECR com grupos de controle ativo para identificar a influência de cada elemento do programa. Por fim, a adesão às práticas formais e informais de *mindfulness* foi medida com base nos relatos dos participantes, o que pode resultar em subnotificação ou supernotificação da prática realizada. Consideramos importante aprimorar as formas de medir a adesão às práticas formais e informais de *mindfulness* e avaliar o perfil das pessoas que aderem a determinadas práticas. São necessárias mais pesquisas para qualificar o protocolo de intervenção atual e identificar os mecanismos subjacentes aos efeitos preliminares positivos observados.

## Conclusões

O Programa *Hemomindful* mostrou-se uma intervenção complementar ao TAU com base nos indicadores positivos de viabilidade e boa retenção, aceitabilidade e segurança. Além disso, foram apresentados resultados qualitativos preliminares sobre o impacto no manejo da dor e do desconforto associados ao tratamento de HD e a adesão a comportamentos relacionados ao tratamento, à saúde e ao bem-estar dos participantes do Programa *Hemomindful*. De acordo com nossos resultados, esses benefícios não apenas auxiliam no manejo da dor, mas também contribuem para melhorar o bem-estar geral durante o tratamento de HD. Se estudos futuros corroborarem esses efeitos, o Programa *Hemomindful* poderá ser útil em um amplo espectro de condições de saúde a fim de ajudar a reduzir o sofrimento relacionado à dor.
